# Emerging Materials for Mixed-Matrix Membranes

**DOI:** 10.3390/membranes11100746

**Published:** 2021-09-29

**Authors:** Chong Yang Chuah, Kunli Goh, Tae-Hyun Bae

**Affiliations:** 1 Singapore Membrane Technology Centre, Nanyang Environment and Water Research Institute, Nanyang Technological University, Singapore 637141, Singapore; chongyang.chuah@ntu.edu.sg (C.Y.C.); gohkunli@ntu.edu.sg (K.G.); 2 Department of Chemical and Biomolecular Engineering, Korea Advanced Institute of Science and Technology, Daejeon 34141, Korea

This Special Issue, entitled “Emerging Materials for Mixed-Matrix Membranes” was introduced to cover the recent progress in the development of materials for mixed-matrix membranes (MMMs) with potential application in fields such as sea water desalination, gas separation, pharmaceutical separation, wastewater treatment and the removal of pathogenic (viruses and bacteria) microorganisms as well as solvents and resource recovery. In general, MMMs utilize a classical strategy to improve membrane performances through leveraging the physicochemical properties of nanomaterials (that serve as filler materials) to tune the molecular transport properties of polymer matrices. In recent years, the choice of nanomaterials with promise as filler materials in polymeric membranes has seen a rapid expansion, considering different potential advantages of each filler category, with the eventual aim of improving the overall separation performances.

MMMs in gas separation have been investigated in several experimental studies. For instance, the applicability of membranes for carbon dioxide (CO_2_) capture has been a focus of attention, considering the fact that the CO_2_ concentration in the atmosphere has surpassed 400 ppm since 2013 [1]. In this regard, a carbon capture and sequestration (CCS) process was proposed as one of the feasible solutions to minimize the emission of greenhouse gas (GHG) from its source, namely, the combustion of fossil fuels for energy generation [2]. Membrane-based technology is set to be an attractive approach for CO_2_ separation compared with other technologies such as amine-scrubbing, which suffers from a high energy penalty. In the study conducted by Chuah et al. [3], the development of mixed-matrix carbon molecular sieve membranes (CMSMs) with the incorporation of carbon-based fillers (i.e., graphene oxide (GO) and activated carbon (YP-50F)) demonstrated this case. Based on the gas permeation data, the incorporation of carbon-based fillers was feasible in improving the gas permeability of mixed-matrix CMSMs to a substantial extent. This corresponded with improvements of 36% and 110% as well as 64% and 202% for 15 wt% GO and 15 wt% YP-50F in carbonized membranes using Matrimid^®^ 5218 and OPDA-TMPDA (4,4’-oxydiphthalic anhydride and 2,4,6-trimethyl-*m*-phenylenediamine) as the polymeric precursors, respectively. The improvements were attributed to the presence of large micropores (>8 Å) in the mixed-matrix CMSM that facilitated the transport of CO_2_ through the membranes.

The applicability of CMSMs has also been investigated in the field of air separation. In contrast to CO_2_-based separation, where CO_2_ permeation is comparatively more favorable in polymeric membranes due to higher CO_2_ solubility and diffusivity compared with N_2_, the separation of oxygen (O_2_) and nitrogen (N_2_) is much more challenging. This is because, in terms of solubility-diffusivity analyses, O_2_ diffusivity and N_2_ solubility are comparatively higher than those of the other gas components [4]. Despite several materials having shown a favorable O_2_ adsorption, such processes are typically irreversible during the regeneration process. Therefore, in the study conducted by Chuah et al. [5], porous zeolite nanocrystals (PS-MFI), which possess a high porosity, were incorporated into the CMSMs with the aim of improving the O_2_ permeability of the membrane. Based on the reported separation performances, the incorporation of 20 wt% and 30 wt% PS-MFI in carbonized Matrimid^®^ membranes was able to push the membrane performances to surpass the upper boundary limit for O_2_/N_2_ separation. Their results indicated that despite the marginal sacrificial decrease in O_2_/N_2_ selectivity, the strong escalation in O_2_ permeability was feasible to reconcile this shortcoming. Moreover, the use of zeolite nanocrystals, which possess a high thermal stability, was critical for the CMSM fabrication due to the need to conduct the carbonization process at a high temperature (550 °C). 

Apart from this, the potential of MMMs for hydrogen (H_2_) separation has been a hot topic recently, owing to climate change and increased opportunities for renewable energies [6]. At present, H_2_ continues to be produced through primary energy resources such as fossil fuels and so efforts in mitigating carbon emissions are still highly desirable as the byproducts from H_2_ generation typically involve N_2_, CH_4_ and CO_2_ [7]. Thus, in the review by Chuah et al. [8], the recent development of MMMs for the separation of different gas pairs involving H_2_ was thoroughly discussed. Several filler categories such as zeolites, metal–organic frameworks (MOFs), covalent–organic frameworks (COFs), graphene-based materials and the selection of binary fillers for potential H_2_ separation were the main highlight of this review. In general, it is well-agreed that H_2_-based membrane separation is challenged by the fast diffusivity and low solubility of H_2_ molecules, leading to substantial difficulties in allowing a clear discrimination of other penetrant gas molecules. The review paper discusses how H_2_ separation can be better achieved through rational designs of the filler materials in the MMMs.

Other than gas separation, membranes are also feasible for use in water treatment processes by removing organic and inorganic pollutants generated from anthropogenic activities [9]. It has been reported by the United Nations that up to 80% of municipal and industrial wastewaters are discharged into the environment without proper treatment. Such actions pose a high risk of environmental harm, which necessitates the need for water treatment intervention at various levels. In terms of the effective treatment of wastewater, the incorporation of nanomaterials into membranes can be a compelling strategy to improve the separation efficiency, as reported in substantial literature studies. Thus, a review on pollutant removal from wastewaters with the utilization of MMMs was thoroughly conducted by Lim et al. [10]. This involved the investigation on the potential applications of the membranes in the removal of heavy metals (arsenic, mercury, cadmium and lead), dyes, humic acid, organic compounds, nitrates and ammonia. However, the utilization of MMMs in the wastewater treatment process is mainly challenged by three main factors. First, the removal of heavy metals is highly specific with several other contaminants only able to meet rejection rates of less than 50%. Second, in line with the Sustainable Development Goals (SDG) of the United Nations, efforts in developing materials in a more environmentally friendly manner should be a point of focus. To address this issue, it is possible to use biomass-converted carbon materials as adsorbents to be incorporated into the membranes for the removal of undesirable pollutants. Lastly, microplastics (MPs), which are one of the most widely discussed pollutants in recent years, have been detected in wastewaters due to an increased discharge of non-biodegradable waste from consumers and manufacturing processes. Thus, considering the lack of an effective removal strategy for MPs, membranes have been proposed as a potential solution to be utilized with the selection of appropriate nanomaterials to allow targeted chemical interactions with chemical functionalities present in MPs.

The applicability of membranes in pharmaceutical separation has also been examined in various literature studies, considering its opportunity to be applied under steady-state operations compared with existing technologies such as crystallization and chromatographic and kinetic separations, which are only limited to operations under a batch (non-continuous) configuration. In particular, a chiral resolution (the separation of racemic compounds into their enantiomers) is critical in pharmaceutical separation as most metabolic activities and enzymatic reactions are only feasible with the use of specific enantiomers in order to achieve the required pharmacological effect. Based on the review by Choi et al. [11], the utilization of enantioselective fillers such as COFs, MOFs, zeolites and porous organic cages (POCs) has been undertaken for tuning the diffusivity and selectivity of the resulting MMMs. It was noted that as membranes are subjected to a continuous flow at the upstream and downstream of the membranes, the overall enantioselectivities of the membranes may decrease over a certain period of time. Thus, as suggested by the authors, potential solutions can come with the use of polymer matrices with an intrinsic microporosity of higher rigidity as well as the fabrication of membranes in denser structural configurations. Overall, these approaches aim to decrease the diffusion rates of non-enantioselective species to improve the overall chiral resolution (i.e., enantioselectivity). Alternatively, the utilization of polymers that possess a reasonable enantiomeric separation capability can be made available with the aid of post-synthetic functionalization. This allows a synergistic effect to be achievable, noting that both the polymer and filler are able to effectuate the separation process. Lastly, the stability of the polymer matrix can be potentially enhanced with the use of the vapor phase infiltration (VPI) technique. This technique creates intertwined networks that minimize the undesirable polymer dissolution by organic solvents or membrane swelling, thus increasing its potential for large-scale MMM fabrications.

Membranes can also serve as filters to remove pathogens (e.g., viruses and bacteria) that are present in the environment such as water and air. This technology possesses substantial competitive advantages compared with chemical treatments, namely, disinfection [12]. This is because the disinfection process typically generates harmful byproducts from the chemicals used as well as increases the potential risks of virus resistance toward the disinfectants, despite showing an effective killing capability earlier on. Membrane filtration in general relies mostly on a selective transport and physical barriers and is able to generate the desired separation results without jeopardizing human health. The incorporation of nanomaterials in polymeric membranes has also been proven to disrupt the viral activities of pathogens by interacting with the surface proteins of the pathogens. In addition, a membrane-based water purification process works by size exclusion (separation based on the size of the pathogens) and adsorption (electrostatic or hydrophobic interaction) to trap harmful microorganisms while allowing clean water to pass through the membranes. For air purification processes, membranes predominantly remove harmful airborne bacteria and viruses by a size exclusion effect, albeit with other mechanisms such as interception, inertial impaction and diffusion. Based on the review conducted by Alayande et al. [13], at the present stage, antiviral protective materials (e.g., masks and membranes) are potential solutions to mitigate viruses that are present in the environment. It is expected that, over time, the accumulation of viral particles on membrane surfaces may compromise the overall viral rejection capacity, thus leading to breakthrough contamination in the clean permeate (air or water) streams. The inappropriate disposal of protective materials, which are often one-time use, has raised major concerns due to potential pollution issues as well as secondary infections toward living organisms. Therefore, it is necessary to develop reusable protective materials that demonstrate a strong rejection of pathogens. Efforts in understanding occupational and environmental health risks of exposure to the nanomaterials that are incorporated into the protective materials should be investigated to minimize undesirable health effects toward human beings and living organisms in the long term. This must include detailed short and long term (eco)toxicological assessments to ensure the safe adoption of antiviral protective materials that are nanoenabled [14].

In conclusion, MMMs are expected to circumvent the current limitations in polymeric membranes, most important of which are the trade-off relationship between permeability and selectivity as well as the comparatively less scalable pure molecular sieve membranes [15]. Despite additional studies still being required to increase the reliability of MMMs toward the industrial separation process, it is of fervent hope that MMMs are able to make more important contributions in a not-too-distant future from now.

## Data Availability

Data sharing is not applicable to this article.
